# A Review of Its Medical Aspects.—IV

**Published:** 1909-03-20

**Authors:** 


### THE POOR-LAW COMMISSION'S REPORT.

### A REVIEW OF ITS MEDICAL ASPECTS.—IV.

MINORITY REPORT.—*Concluded.*

*The Effect of the Public Health Medical Service on its Patients.*—There is absolute uniformity of testimony, from all sorts of witnesses in all parts of the country, that the medical attendance and medicine of the Public Health Department has no pauperising tendency. The fact that the services are rendered in the interest of the community seems actually to create in the recipient an increased feeling of personal obligation, and even a new sense of social responsibility.

The Public Health medical service, as it exists to-day, has grave defects. Over a large part of the country it exists only in skeleton outline. A majority of the 650 rural Sanitary Authorities, and not a few of the smaller urban Sanitary Authorities have no hospital even for the most infectious diseases, no system of domiciliary visitation, and but a scrap of the time of a private practitioner for a medical officer of health. Even in many considerable urban districts isolation hospitals exist only for three or four of the chief zymotics. The apathy of the Local Authorities is not systematically exposed by any regular inspection by the Local Government Board, while zeal and enterprise appear to meet with little official recognition and encouragement. The varied activities described have, in fact, often emanated from the zeal of the medical officer of health himself. In short, the Public Health medical service, though excellent in its aims and results, and demonstrably successful in a few zealous districts, for such diseases as it has there touched, is, from a national standpoint, suicidally deficient in its volume and geographical extension.

(h) *The Need for a United Medical Service.*

In consequence of overlapping and confusion, the Poor-law and Sanitary Authorities are spending as much as seven or eight millions sterling annually on the curative treatment of the sick, and between them fail to provide for a large proportion of the illness—even of the preventable illness of the community. The proportion of uncertified deaths, indicating a total lack of medical attendance even in the most advanced stages of disease, amounts in certain towns in England to 4 or 5 per cent., in certain counties of Scotland to 20 and even 30 per cent., in some islands to as many as 60 or 70 per cent. An enormous amount of incipient disease exists—undiscovered, untreated, and unchecked. The Poor-law Medical Service, with its stigma of pauperism, its deterrent tests, and its consequent failure to get hold of incipient disease is, from the standpoint of national health (at any rate in a large proportion of cases), practically useless. If the district medical officer dispenses physic rather than advice, it positively counteracts the promotion of personal hygiene; if he orders "medical extras" he introduces the patient to reliance on the food or money dealt out by the relieving officer. The existence of the Poor-law Service gives the Local Health Authority an excuse for not acting upon what are actually its statutory powers. The medical practice of the united Public Medical Service must be based upon Public Health rather than upon Poor-law principles, and the imperative need for unifying the present competing services is felt by the heads of all the four public departments concerned.

*Conclusions.*—We have therefore to report :—

1. That the continued existence of two separate rate-supported medical services in all parts of the United Kingdom, costing, in the aggregate, six or seven millions sterling annually—overlapping, unco-ordinated with each other, and sometimes actually conflicting with each other's work—cannot be justified.

2. That the very principle of the Poor-law Medical Service—its restriction to persons who prove themselves to be destitute—involves delay and reluctance in the application of the sick person for treatment; hesitation and delay in beginning the treatment; and, in strictly administered districts, actual refusal of all treatment to persons who are in need of it, but who can manage to pay for some cheap substitute. These defects, which we regard as inherent in any medical service administered by a Destitution Authority, stand in the way of the discovery and early treatment of incipient disease, and accordingly deprive the medical treatment of most of its value.

3. That it has been demonstrated to us beyond all dispute that the deterrent aspect which the medical branch of the Poor Law acquires through its association with the Destitution Authority, causes, merely by preventing prompt and early application by the sick poor for medical treatment, an untold amount of aggravation of disease, personal suffering, and reduction in the wealth-producing power of the manual working class.

4. That the operations of the Poor-law Medical Service, being controlled by Destitution Authorities and administered by destitution officers, inevitably take on the character of unconditional "medical relief"—that is, relief of the real or fancied painful symptoms—as distinguished from remedial changes of regimen and removal of injurious conditions, upon which any really curative treatment, or any effective prevention of the spread or recurrence of disease, is nowadays recognised to depend.

5. That while domiciliary treatment of the sick poor is appropriate in many cases, it ought to be withheld (1) where proper treatment in the house is impracticable; (2) where the patient persistently malingers or refuses to conform to the prescribed regimen; or (3) when the patient is a source of danger to others. It has become imperative in the public interest that there should be, for extreme cases, powers of compulsory removal to a proper place of treatment. Such powers cannot, and in our opinion should not, be granted to a Destitution Authority.

6. That where Destitution Authorities cease to abide by the limitation of their work to persons really destitute, or pass beyond the dole of "medical relief," their attempt to extend the range or improve the quality of the Poor-law Medical Service brings new perils. We cannot regard with any favour any action which, in order to promote treatment, opens, or tacitly invites, people voluntarily to range themselves among the destitute; or which tempts them, by the prospect of getting costly and specialised forms of treatment, to simulate destitution. Nor do we think that an authority charged with the relief of destitution, whatever its method of appointment, or whatever the area over which it acts; or any authority acting through officers concerned with such relief, whatever their official designation, can ever administer a medical service with efficiency and economy.

7. That, with regard to the suggestion that the medical treatment of the sick poor should be left either to provident medical assurance or to voluntary charity, it has been demonstrated to us that these offer no possible alternative to the provision for the sick made by the public authority. With regard to domiciliary treatment, the evidence as to medical clubs, "contract practice," provident dispensaries, and the out-patient departments of hospitals, is such as to make it impossible to recommend, in their favour, any restriction of the services at present afforded by the district medical officers and Poor-law dispensaries. Nor do we feel warranted in giving any support to the proposal made to us that the whole of the outdoor medical service of the Poor Law should be superseded by a publicly-subsidised system of letting the poor choose their own doctors. Any such system would, in our judgment, lead to an extravagant expenditure of public funds on popular remedies and "medical extras," without obtaining in return for this enlarged "medical relief," greater regularity of life or more hygienic habits in the patient. With regard to institutional treatment, we gladly recognise the inestimable services rendered to the sick poor by the hospitals, sanatoria, and convalescent homes supported by endowments or voluntary contributions. We approve of the use now made of these institutions by public authorities, and we think that many more suitable cases than at present might, on proper arrangements as to payment, be transferred from rate-maintained to voluntary institutions. But it is clear that such institutions provide only for a small fraction of the need, and that they leave untouched whole districts for some cases, and whole classes of cases everywhere, which there is no prospect of their being able to undertake.

8. That the medical service of the Public Health Authorities, which now extensively treats disease, and actually maintains out of the rates a steadily increasing number of the sick poor, is based on principles more suited to a State medical service than that of the Poor Law. These principles, which lead in practice as well as in theory to searching out disease, securing the earliest possible diagnosis, taking hold of the incipient case, removing injurious conditions, applying specialised treatment, enforcing healthy surroundings and personal hygiene, and aiming always at preventing either recurrence or spread of disease in contrast to the mere "relief" of the individual—furnish, in fact, the only proper basis for the expenditure of public money on a medical service.

9. That such compulsory powers of removal in extreme cases, as have been asked for, are analogous to those already exercised, with full public approval, by the Public Health Authorities; and that the proposed extension of such powers can properly be granted only to an authority proceeding on Public Health lines.

10. That we therefore agree with the responsible heads of all the four Medical Departments concerned . . . in ascribing the defects of the existing arrangements fundamentally to the lack of a unified medical service based on Public Health principles.

11. That in such a unified medical service, organised in districts of suitable extent, the existing medical officers of health, hospital superintendents, school doctors, district medical officers, workhouse and dispensary doctors, and medical superintendents of Poor-law infirmaries, the clinicians as well as the sanitarians would all find appropriate spheres; that one among them being placed in administrative control who has developed most administrative capacity.

12. That we do not agree with the suggestion that the establishment of a unified medical service on Public Health lines necessarily involves the gratuitous provision of medical treatment to all applicants. It is clear that in the public interest, neither the promptitude nor the efficiency of the medical treatment must be in any way limited by considerations of whether the patient can or should repay its cost. But we see no reason why Parliament should not embody, in a clear and consistent code, definite rules of chargeability, either relating to the treatment of all diseases or of all but those specifically named, and of recovery of the charge thus made from all patients who are able to pay.

H. Russell Wakefield. George Lansbury.

Francis Chandler. Beatrice Webb.

### MEMORANDUM BY DR. DOWNES ON THE REPORTS OF THE COMMISSION.

Dr. Downes signed the Majority Report to support the principle of one Local Authority in each area for public relief in every form. He considers the scheme of administration proposed in the Majority Report to be unworkable and dangerous. A proposal to sweep away all directly elected representation requires most rigid proof of its necessity; every statement should be carefully tested, and all contingent conditions taken into account; but the extracts from the evidence given, even in the Majority Report, do not sufficiently comply with these requirements in all cases. Considerable advances have been made and continue to be made in the administration of the Poor Law—*e.g.* in classification, 38 per cent. of the indoor poor being already provided for in specialised institutions; and it is well to remember that classification run to an ideal entails many practical difficulties and even evils.

Every defect mentioned could be better met by a revision, a strengthening and extension of existing powers on lines already established. The area of administration proposed would entail 225 adjustments *without finality being secured*, owing to the growth of urban districts and boroughs, and in some cases the present area would be reduced. Ninety-two Poor-law Unions have each a population of over 100,000; 77 only of the counties and county boroughs have such a figure. The grouping of county and county borough areas would be difficult or practically impossible, and thus the problem of the fringe of the great cities would remain. From these and other objections a policy of grouping or amalgamating existing Unions would be largely free. A strong control of public relief is a vital necessity, and should be in the hands of one authority. Committees, even of the same body, are usually jealous of each other, and in all probability the scheme of the Majority would only be a halfway step to the multitude of relief authorities advocated by the Minority.

Much may be done for the sick by nursing organisations and by the development of county infirmaries and cottage hospitals under the auspices of the Voluntary Aid Councils and Committees, proposed by the Majority; but on these Councils and Committees the medical profession should be represented. The Majority scheme for medical relief is too complicated for adoption in practice, and offers what amounts to a large measure of free medical relief without adequate safeguard either to the medical profession generally or to the ratepayer. For co-operation with the sanitary services, as recommended by the Minority, provision already exists and could be developed without difficulty. Any amalgamation of the two services would be detrimental and contrary to the public interest.

Disfranchisement should, at any rate, be reserved for certain cases—*e.g.* persons able to pay but failing to repay for infirmary relief obtained by them. "At present a man is not disfranchised if he gets relief which he ought to have been able to provide for himself. He may be disfranchised for seeking relief he could not reasonably be expected to provide, and which, in addition, it was often to the public benefit as well as his own that he should get."

The whole question of the cost of hospital and infirmary construction and administration and the standard of equipment should be inquired into by a Departmental Committee, and a strong Statistical Department and Intelligence Bureau established as a division or branch of the Central Authority.

---

"The Hospital" Pension for 1909 in connection with the Newsvendors' Benevolent and Provident Institution has been awarded by the Committee to Charles Skeats.

